# Electrolyte Magnetohydrondyamics Flow Sensing in an Open Annular Channel—A Vision System for Validation of the Mathematical Model

**DOI:** 10.3390/s18061683

**Published:** 2018-05-24

**Authors:** Mónica Valenzuela-Delgado, Wendy Flores-Fuentes, Moisés Rivas-López, Oleg Sergiyenko, Lars Lindner, Daniel Hernández-Balbuena, Julio C. Rodríguez-Quiñonez

**Affiliations:** Facultad de Ingeniería, Universidad Autónoma de Baja California, Mexicali C.P. 21280, Baja California, Mexico; monicavd74@uabc.edu.mx (M.V.-D.); mrivas@uabc.edu.mx (M.R.-L.); srgnk@uabc.edu.mx (O.S.); lindner.lars@uabc.edu.mx (L.L.); dhernan@uabc.edu.mx (D.H.-B.); julio.rodriguez81@uabc.edu.mx (J.C.R.-Q.)

**Keywords:** magnetohydrodynamics, sensing, microfluidics, vision system

## Abstract

Magnetohydrodynamics (MHD) is becoming more popular every day among developers of applications based on microfluidics, such as “lab on a chip” (LOC) and/or “micro-total analysis systems” (micro-TAS). Its physical properties enable fluid manipulation for tasks such as pumping, networking, propelling, stirring, mixing, and even cooling without the need for mechanical components, and its non-intrusive nature provides a solution to mechanical systems issues. However, these are not easy tasks. They all require precise flow control, which depends on several parameters, like microfluidics conductivity, the microfluidics conduit (channel) shape and size configuration, and the interaction between magnetic and electric fields. This results in a mathematical model that needs to be validated theoretically and experimentally. The present paper introduces the design of a 3D laminar flow involving an electrolyte in an annular open channel driven by a Lorentz force. For an organized description, first of all is provided an introduction to MHD applied in microfluidics, then an overall description of the proposed MHD microfluidic system is given, after that is focused in the theoretical validation of the mathematical model, next is described the experimental validation of the mathematical model using a customized vision system, and finally conclusions and future work are stated.

## 1. Introduction

The application of magnetohydrodynamics (MHD) in microfluidics represents a new trend during the last twenty years. Micro-applications started to develop and surged with the need to propel, stir, and control flows to perform tasks such as mixing and networking at the micro-scale. They have been particularly useful for microfluidic applications where a conductive fluid needs to be manipulated to perform specific operations. The progress achieved in this multidisciplinary field and its incursion into a big variety of applications can be followed through the scientific literature. Nevertheless, it still remains as an area for research that requires further development [1]. Its principle of operation is based on the Lorentz force acting in an electrical conductive solution, in the presence of magnetic and electric fields, resulting in a motion flow. The most used solutions in the beginnings of MHD research were liquid metals and ionized gases, though in the more recent scientific literature electrolyte solutions have made an incursion [2]. This is because the flow in a MHD stirrer depends on the solution electrical conductivity. Electrolytes are a convenient option, even though a nonlinear electrode kinetics phenomenon occurs due to the use of electrolytes. The electrolyte flow is usually contaminated with electrochemical reactions occurring at the electrodes surfaces [3]. On the other side, the hydraulic properties and conductivity of the MHD flow depends of the channel geometry [4] (shape, size, proportions, etc.) and the electrode arrangement. While rectangular conducts are the most common, i.e., two parallel electrodes set on two opposing walls [5], there also exist other configurations with annular channels, where the channel is formed between an internal and an external electrode [6]. Another aspect to consider is that channels completely closed and filled with solution exclude the presence of free surfaces and as consequence display surface tension effects, while others are open channels, with a free surface. Regarding magnetic and electric fields they can be induced by permanent magnets, electromagnets, DC or AC power supplies [7,8,9]. Different MHD system configurations can be found in the scientific literature over the last few years, opening up a wide range of applications. One example of an MHD system with a novel configuration is presented in [2], which accomplished a mixing enhancement by the creation of a turbulent-like chaotic advection. The chaotic advection has been achieved thanks to application of an AC voltage across specific disk electrodes and a ring counter electrode switching on and off a pair of disk electrodes with a certain period T, producing a “blinking vortex”.

The inclusion of microfluidics in the design of micro-electromechanical systems (MEMS) provides an alternative solution to mechanical friction, thanks to the advantage of its non-intrusive nature. The lack of mechanical elements reduces the possibilities of breakdowns, undesired vibrations and noise. A direct transformation from electromagnetic to mechanical energy is performed for all those applications that require accuracy in micro-scale systems [10]. The most common applications of MHD are micro-pumping (in labs-on-chip—LOCs—and liquid chromatography (LC)) [8,11,12], networking (in LOCs) [8], stirring and mixing (in LOCs, polymerase chain reaction (PCR), and chemical reactors) [2,13,14], micro-cooling (in microelectronics), and thermal cyclers [3]. Summaries of the most recently MHD-based microfluidics devices are given in [11,15]. The present paper introduces the design of a 3D laminar electrolyte flow produced in an annular open channel driven by a Lorentz force. A theoretical and experimental study has been done as explained below. The theoretical research consisted of: (a) the study of the state of the art of annular channels driven by a Lorentz force, (b) the development of the mathematical model, (c) the velocity steady flow quasi-analytic approximation by a Galërkin method with orthogonal Bessel-Fourier series, and (d) the validation of the model quasi-analytic approximation versus other models resolved using: (i) analytic solution (AS) [16] and (ii) finite element method (FEM) methods [17]. Galërkin methods have been used before with a numerical approach in other research, resulting in an accurate and excellent method to be combined with other mathematical methods. A review of the mathematical methods applied to the different MHD configurations can be found in [6]. The experimental research consisted in: (a) the fabrication of an MHD prototype, an annular channel formed by two concentric electrical conducting cylinders limited by an insulating bottom wall, (b) the development of a customized vision system for the sensing of the velocity profiles in an electrolyte driven in azimuthal direction by a Lorentz force induced by the interaction between a uniform magnetic field along the axial direction and a radial electric current, (c) the mathematical model validation in order to characterize the effect of the three walls implicated in the MDH prototype considering the gap dimension between the cylinders as well as the depth of the channel. The MHD overall microfluidic system is presented in Section 2. Section 3 describes the theoretical research activities done to validate the proposed mathematical model. The customized vision system and the velocity profiles sensing methodology are described in Section 4 for the experimental validation of the 3D laminar electrolyte flow produced in the MHD prototype. Finally, conclusions and future work directions are provided.

## 2. MHD Microfluidic System Overall

**Figure 1:**
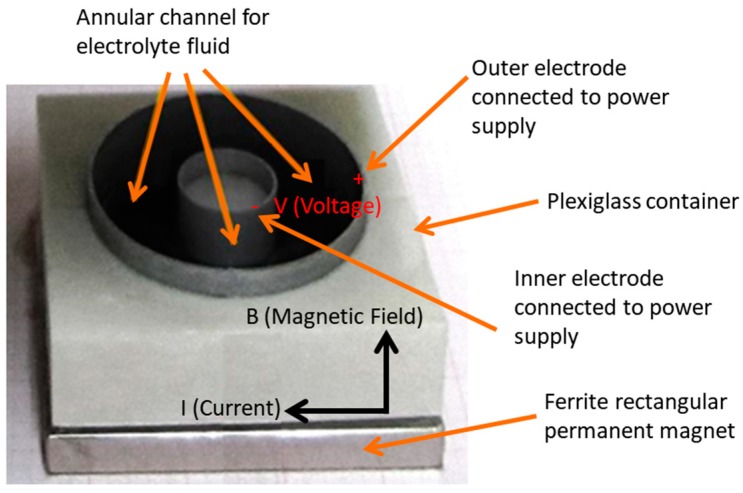
Geometric shape and elements of the MHD microfluidic system (MHD stirrer).

The proposed design of an MHD stirrer with a 3D laminar electrolyte flow in an annular open channel driven by a Lorentz force is shown in Figure 1. The MHD stirrer consists of an annular channel of depth (h=7 mm) formed with the gap between two electrical conducting cylinders with radios $R_{1}$ (18.76 mm) and $R_{2}\left(31.44\;\mathrm{mm}\right)$ made of copper (1 mm of thickness) acting as +/− electrodes, with an insulating bottom wall of 10 × 10 cm made of passive self-peeling (PSP) resin from a 3D print, and a free surface (container without top wall).

The annular channel is centered in a neodymium permanent magnet (SMBN0643 Stanford-Magnet, Lake Forest, CA, USA). The permanent magnet induces a uniform magnetic field $\left(B_{0}=0.1624\;T\right)$ parallel to the cylinders axes (in vertical direction). When an electric potential difference (ϕ, DC voltage) is applied between the cylindrical electrodes of copper, a radial electric current (I) arises in the electrolyte fluid. An azimuthal Lorentz force is produced by the interaction of the radial current with the axial magnetic field. The azimuthal Lorentz force drives the flow generating a distributed body force on the fluid. In consequence, a continuous circulation of fluid in a closed loop is produced, obtaining a channel with a virtual infinite length. If current flows inwardly (outwardly) the fluid moves in the counter-clockwise (clockwise) direction.

The selected microfluidic is an electrolyte (distilled water seeded with 10 nm silver-coated hollow glass spheres, seeding particles). The seeded particles make the distilled water electrically conductive and less vulnerable to bubble generation and electrode oxidation. Due to electrolytes are fluids of low conductivity with uniform electric conductivity (σ), mass density (ρ), and kinematic viscosity (v) allow propel them without inducing a change in the applied electric field. By consequence, a microfluidic behavior is obtain with a velocity uncoupled from the potential difference, and with an induced current that does not add drag forces, which could affect the flows configuration. Besides, the heating effects due to current fluxes transmitted through the electrolyte are negligible.

## 3. Theoretical Validation of Mathematical Model

The mathematical model of an electrolyte conducted by MHD flow in an open annular channel and its validation is presented in this section. In open channels with a finite depth, the presence of the bottom wall influences the flow configuration, acting like an additional drag force in the fluid, also the cylinders walls produce particular effects that determine the flow behavior and influence its stability. Closed channels have been analyzed previously, taking into account the effects of the four walls [15], however this analysis is focused on open channels. In an open channel, the effect of the walls can be transmitted to the flow in the free surface on the top of the channel.

### 3.1. Mathematical Formulation of the Problem and Governing Equations

A 3D steady laminar flow of an electrolyte in an open annular channel driven by an azimuthal Lorentz force is analyzed. The 3D steady laminar flow analysis is performed in a cross-section of the open channel. And the effect of the walls is considered during the analysis. Walls provide a type of friction, which produces a fluid resistance, commonly called drag force. Drag forces act opposite to the fluid motion; they can appear on fluid surfaces with a magnitude proportional to the velocity of the laminar flow. The effect of the three walls is analyzed in detail by considering the gap between the cylinders as well as the depth of the channel. Since the magnetic field induced by the electric currents circulating in the fluid is much smaller than the applied magnetic field, the magnetic Reynolds number is very low $\left(R_{m}\ll 1\right)$. Consequently, the continuity and the Navier-Stokes equations (that usually are used to govern the MHD flow in annular ducts) in conjunction with Ohms law and the equation for electrical potential (that manifest the charge conservation) can be neglected. As the microfluidic is an electrolyte, the Hartmann number is very small (Ha≈0.01), resulting in the decoupling of velocity and electric potential parameters. The exact solution for azimuthal velocity $\left(u_{\theta }\right)$ implicit in (1) can be determinate solving the Laplace equation $\nabla^{2}\phi =0$ applying the boundary conditions: (a) the lateral conductive walls located at r=η, and r=1, are given by (2), where $\eta =R_{1}/R_{2}$. (b) the bottom insulating Hartmann wall and the free surface on the top are located at z=0, and z=1 given by (3) and (4) respectively, while $\varepsilon =h/R_{2}$ is the aspect ratio, which is affected by the height of the channel:

(1) $$\frac{\partial^{2}u_{\theta }}{\partial r^{2}}+\frac{1}{r}\frac{\partial u_{\theta }}{\partial r}-\frac{u_{\theta }}{r^{2}}+\frac{1}{\varepsilon^{2}}\frac{\partial^{2}u_{\theta }}{\partial z^{2}}-\frac{1}{r\left|\ln\eta \right|}=0$$

(2) $$u_{\theta }\left(r,z\right){|}_{r}=0$$

(3) $$u_{\theta }\left(r,z\right){|}_{z=0}=0$$

(4) $$\frac{\partial \;u_{\theta }\left(r,z\right)}{\partial z}{|}_{z=1}=0$$

The approximating solution of the non-homogeneous differential Equation (1) subject to conditions (2)–(4), is obtained substituting Bessel functions of first and second order and satisfying the boundary conditions using eigenvalues. On the other hand, the flow rate (q) expressed by (5) provides the analytical results obtained using Galërking approximation method, where $v_{\theta }$ is the approximate solution for azimuthal velocity:

(5) q=∫−1212∫η1vθdrdz

In order to reduce the amount of time the technical computing software requires for solving the equations the velocity and flow rate equations were made dimensionless. The characteristic velocity $\left(U_{c}\right)$ and characteristic flow rate $\left(Q_{c}\right)$ were expressed using the current parameter (I) as a control parameter, resulting in (6) and (7) respectively, where (μ) represents the magnetic permeability. Finally, the characteristic average velocity $\left({\bar{U}}_{AVG}\right)$ by (8) has been established, a product of the division of the flow rate between the area of the channel cross-section:

(6) $$U_{c}=\frac{R_{2}\sigma \Delta \phi B_{0}}{\mu }=\frac{R_{2}IB_{0}\left|\ln\eta \right|}{2\pi h\mu }$$

(7) $$Q_{c}=\frac{R_{2}^{2}\sigma h\Delta \phi B_{0}}{\mu }=\frac{R_{2}^{2}IB_{0}\left|\ln\eta \right|}{2\pi \mu }$$

(8) $${\bar{U}}_{AVG}=\frac{R_{2}\sigma \Delta \phi B_{0}}{\left(1-\eta \right)\mu }=\frac{R_{2}IB_{0}\left|\ln\eta \right|}{2\left(1-\eta \right)\pi h\mu }$$

After applying the non-slip boundary condition, the eigenvalues of the Bessel functions are determined for each η value. Obtaining a velocity and a flow rate solutions expressed by (9) and (10) correspondingly. Different values of the aspect ratio ε obtained according with the η used value:

(9) $$v_{\theta }=\overset{M}{\underset{m=1}{\sum }}\overset{N}{\underset{n=1}{\sum }}\frac{8{\left(-1\right)}^{n+1}\alpha_{m}}{\left(\alpha_{m}^{2}+\frac{{\left[\left(2n-1\right)\pi \right]}^{2}}{4\varepsilon^{2}}\right)\left(v_{1;m}^{´2}\left(1\right)-\eta^{2}v_{1;m}^{´2}\left(\eta \right)\right)\left|\ln\eta \right|}\frac{v_{0;m}\left(1\right)-v_{0;m}\left(\eta \right)}{\left[\left(2n-1\right)\pi \right]}v_{1,m}\left(r\right)g_{n}\left(z\right)$$

(10) $$q=-\overset{M}{\underset{m=1}{\sum }}\overset{N}{\underset{n=1}{\sum }}\frac{16}{\left(\alpha_{m}^{2}+\frac{{\left[\left(2n-1\right)\pi \right]}^{2}}{4\varepsilon^{2}}\right)\left(v_{1;m}^{´2}\left(1\right)-\eta^{2}v_{1;m}^{´2}\left(\eta \right)\right)\left|\ln\eta \right|}\frac{{\left(v_{0;m}\left(1\right)-v_{0;m}\left(\eta \right)\right)}^{2}}{{\left[\left(2n-1\right)\pi \right]}^{2}}$$

### 3.2. Computing Results of the Mathematical Model

**Figure 2:**
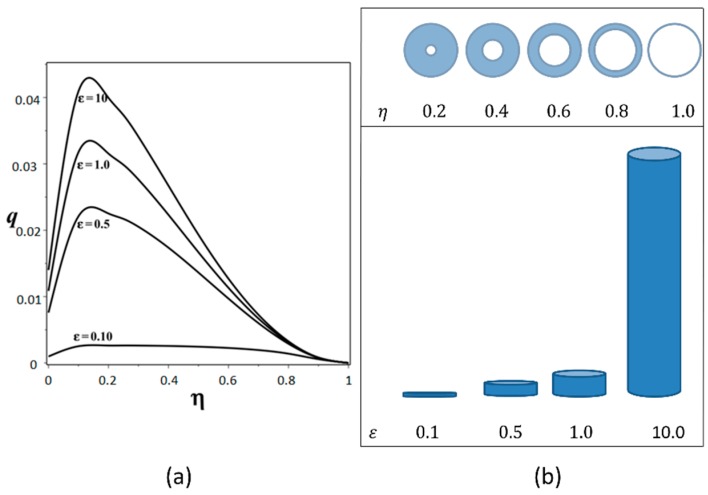
(**a**) 2D dimensionless flow rate *q* versus *η* for aspect ratio *ε* = 0.1, 0.5, 1.0, 10.0. (**b**) Physical representation of *η* and *ε* relation.

**Figure 3:**
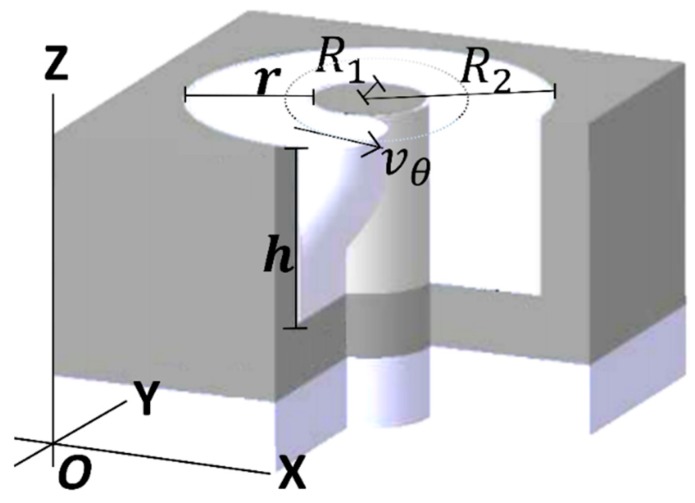
Annular channel cross-section and its main parameters: h (channel depth), r (channel width), $R_{1}$ (inner cylinder radius), $R_{2}$ (external cylinder radius), $v_{\theta }$ (azimuthal velocity).

The mathematical model was simulated using Maple [18]. With the intention to analyze the fluid behavior for different values of parameters, 3D numerical simulations were used with several parameter configurations. The dimensionless flow rate expressed by (10) has been evaluated for η=[0.2:0.2:1.0] with ε=0.1,0.5,1.0,10.0 and Figure 2 illustrates this simulation. The dimensionless flow rate q increases directly proportional to the ε increase for any given η value. However, due to existing friction at the walls, the flow rate q is reduced as ε takes smaller values, as well as when the gap becomes zero (η → 1). For the different values of ε the maximum dimensionless flow rate is at η=0.15. By other side, ε = 10 can be considered as a limit since for higher values the flow rate q does not change dramatically. The annular channel cross-section is shown in Figure 3, it shows the main parameters: internal cylinder radio ($R_{1}$), external cylinder radio ($R_{2}$), channel depth (h) in the Z axe, dimensionless channel width (r), and velocity profile ($v_{\theta }$) through length r.

**Figure 4:**
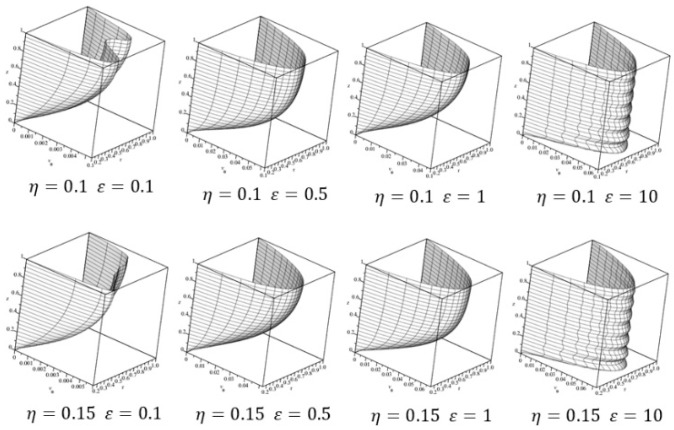
3D dimensionless velocity profiles for several parameter configurations, in order to show the symmetry and anti-symmetry of the different parameter configurations.

Velocity profiles are explored by varying the channel depth (h) and the gap between the cylinders in order to show the effect of the walls on the flow behavior. In Figure 4 the velocity profiles for *η* = 0.1 and *η* = 0.15 with aspect ratios *ε* = 0.1, 0.5, 1.0, 10.0 (several depths of the channel) are illustrated. For a small aspect ratio, the velocity profile on the surface is anti-symmetric due to the effect of the walls. However, for a high aspect ratio, the velocity profile on the surface tends to be symmetric due to the surface is almost not affected by the bottom wall. Also, the symmetric profile can be reached as the internal radio approaches to the external radio.

### 3.3. Comparison of the Proposed Mathematical Model with Other Models Found in Previous Research Literature

During the research of the state of art, was found that previous experiments have been done where a circular flow of a weakly conducting fluid was induced by a Lorentz force. Nevertheless, most of them assume that cylinders are infinitely long (ε→∞) and that viscous friction is not present due to the non-existence of bottom effects [19].

Although in a few studies, cylinders are finite [20], and a theoretical Q2D model (quasi-two-dimension model) has been developed for a circular channel with an upper free surface with a very thin fluid layer (ε=0.1). For the Q2D model analytical solution the friction due to the insulating bottom wall has been considered, with two different conditions: (a) idealized assuming that the fluid does not adhere to the circular channel walls, slip boundary condition $\left(l_{s}\to \infty \right)$, and (b) non-slip boundary condition ($l_{s}$ variying between 0 and 100). The Q2D model was based on an averaging procedure in the normal direction due to the pure viscous friction force present in the fluid caused by the bottom wall. In the 2D velocity profile, it has been found that the magnitude of velocity is almost 50% higher when using weakly conducting fluids, compared to highly conducting fluids, which is due to the Hartmann friction presented in high conducting fluids is stronger than the viscous friction presented in weakly conducting fluid. It has been found, that maximum velocity values are closer to the inner cylinder, although as $R_{m}$ grows, the velocity increases while the profile presents a more parabolic shape. Besides, in spite of the viscous friction at the bottom, the flow rate grows with $R_{m}$ while, and as expected, a substantial gain was also observed as the parameter $l_{s}$ increases. It was observed that for $l_{s}\to \infty$, the flow rate grows linearly with $R_{m}$, until reaching a saturation value.

**Figure 5:**
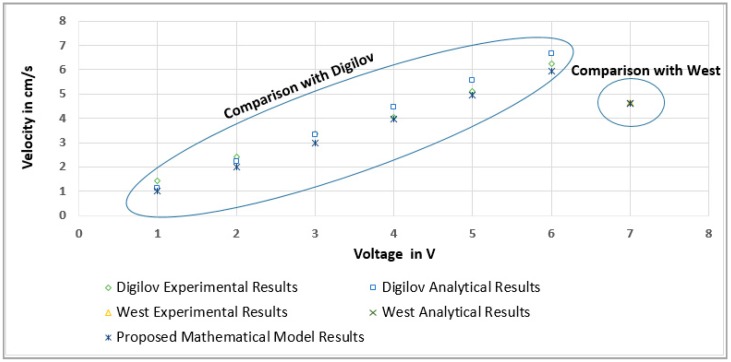
Digilove [16] and West [17] Analitycal and Experimental Results vs. Proposed Mathematical Model Results.

Under these circumstances, it was necessary to develop a 3D model to simulate the velocity profiles. For this reason a model has been developed in [15], based on a quasi-analytic approximation using Galërkin method with orthogonal Bessel-Fourier series, to analyze a closed circular channel, with finite length, taking into consideration a general slip condition on the four walls (top, bottom, inner and external cylinders). This new study has taken into consideration a new shape configuration, that is a circular flow with finite long channel and with free surface (open channel) influenced by the three walls slip conditions (bottom and two sides), with a weakly conducting fluid induced by a Lorentz force. The theoretical validation of the proposed model was performed making a comparison of mathematical model values obtained versus data from previous scientific works with similar configurations:

(a) Analytic Solution (AS) by Digilov [16];

In this research, the experiment was executed in an open channel with a depth of (h=18 mm), formed with the gap between two electrical conducting cylinders of radios $\left(R_{1}=1.5\;\mathrm{mm}\right)$ and $\left(R_{2}=24\;\mathrm{mm}\right)$, with ration (η=0.0625 and ε=0.75), filled with a 2% aqueous solution of CuSO_4_, providing a fluid conductivity $\left(\sigma =0.75\;{\mathrm{Sm}}^{-1}\right)$. Induced by a uniform magnetic field $\left(B_{0}=20\;m\;T\right)$, with Hartmann number $\left(H_{a}=0.01\right)$, and a DC voltage (0.5 V≤ϕ≤3.0 V). Flow patterns visualization was done using a technique based on a liquid dye injection. The liquid dye is dropped horizontally into the channel, using the time scale for the dye, proportional to the magnetic response timescale. The experimental measurement of flow velocity was done using tracking of passive lycopodium dust sprinkled on the free liquid surface. The velocity was measured by counting the number of revolutions completed by a particle within a given amount of time. The flow velocity was modeled by an analytic solution in 2D analysis, obtaining the radial distribution of the azimuthal velocity vector by (11). It was concluded that for any given channel geometry, the velocity is proportional to the applied voltage. Digilov’s experimental and analytical results of this experimentation can be seen in Figure 5, in comparison with the proposed mathematical model:

(11) vθ(r)=σϕB02v ln(1η)r{ln(R2r)+ln(1η)(1η)2−1[1−(R2r)2]}

(b) Finite Element Method (FEM) by West [17];

In this research, a parabolic velocity profile has also been observed for a laminar flow in an MHD annular channel driven by a Lorentz force. A stirrer prototype has been designed for micro-mixing tasks and was characterized using latex particles, fluorescein dye, and a video microscopy system. The time was monitored in which the bromophenol blue and hydrogen ions soluted for each range of velocities. The experiment was executed in an open channel with a depth of (h=500 μm), formed with the gap between two electrical conducting cylinders of radios $\left(R_{1}=4\;\mathrm{mm}\right)$ and $R_{2}\left(6\;\mathrm{mm}\right)$ $\left(R_{1}<R_{2}\right)$, with ration (η=0.667 and ε=0.083), filled with an aqueous solution doped with KCl (1 M) as the charge carrier and 20 particles, providing a fluid viscosity $\left(v=0.9\times 10^{-3}\;{\mathrm{kg}\;m}^{-1}\;s^{-1}\right)$. Induced by an uniform magnetic field $\left(B_{0}=6.6\;m\;T\right)$, and an electric field from an alternating signal $\left(I_{RMS}=85.5\;\mathrm{mA},\;V_{RMS}=6.5\;V,\;f=1\;\mathrm{kHz}\right).$ The mathematical model for this annular MHD propulsion consists in a reduced Navier-Stokes equation with Lorentz force, giving circular streamlines and allowing to reduce the model to a Poisson equation, which is solved using a two dimensional fine element package. This results in a parabolic velocity profile, with a maximum towards the inner wall, at half depth. It was observed that for a given electrode voltage the values of the current (I) changes proportionally with channel height (h) to maintain the current density. Model prediction by FEM was compared with experimental observed velocity and with the proposed mathematically model computing. Figure 5 shows the similarity between models.

## 4. Experimental Validation of the Mathematical Model

The MHD microfluidic system described in Section 2 allows the application of Particle Image Velocimetry (PIV) and/or Particle Tracking Velocimetry (PTV). These techniques were used to perform the experimental validation of the proposed mathematical model, which allow measuring of the flow velocity vectors [21], through the calculation of speckles displacement formed by particles seeded in the flow [22,23]. Since the stirrer configuration facilitates the observation of the free surface over the annular open channel top view side, a customized vision system has been designed and set up to validate the proposed mathematical model, obtaining satisfactory results as detailed below.

### 4.1. Vision System

**Figure 6:**
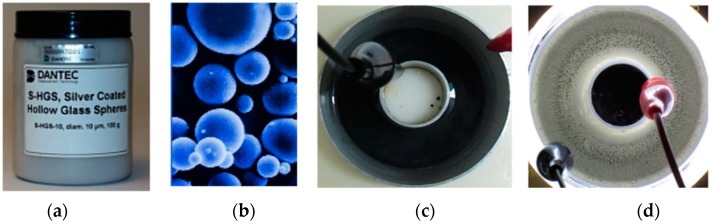
(**a**) Commercial packing of S-HGS particles, (**b**) S-HGS particles, (**c**) Pure electrolyte (**d**) Electrolyte seeded with S-HGS particles forming speckles.

Machine vision systems are widely used for different applications [24]. The proposed machine vision system represents a customized system. It has been integrated into the designed stirrer to measure the electrolyte MHD flow [25]. It contains an illumination subsystem and a camera mounted on a mobile arm over the stirrer. The transparency of the electrolyte allows exploring its flow through the vision system when the fluid has been doped with a high density of particles (forming speckles). The particles selected are silver-coated hollow glass spheres (S-SHGS), borosilicate glass spheres with a smooth and reflectivity surface due to a thin silver coating, as shown in Figure 6.

**Figure 7:**
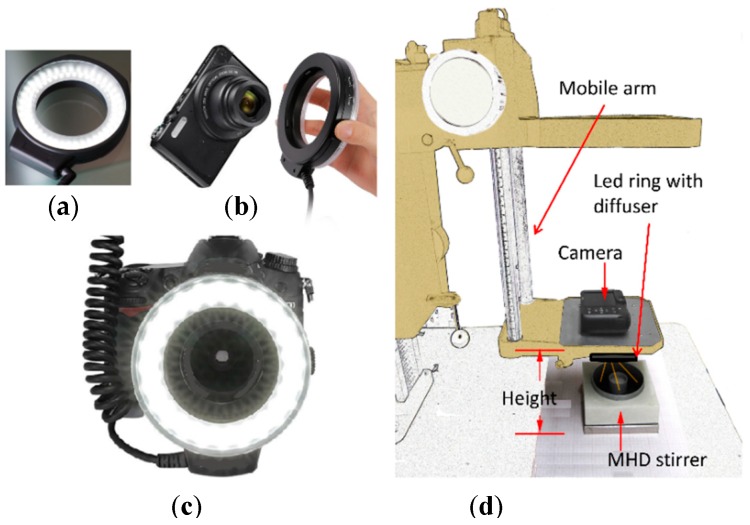
(**a**) LED ring wrapped with a diffuser, (**b**) Camera-LED ring wrapped with a diffuser assembly, (**c**) Dark field illumination sub-system (**d**) Vision system.

The control of the illumination level and the lighting direction play an important role in the video recording performance [21]. By this reason, the free surface of the stirrer flow is illuminated with a LED ring wrapped in a diffuser, to obtain a dark field illumination phenomenon. In this way an undesirable light reflection is avoided. This kind of illumination provides a high contrast image due to the light is guided at the same angle that the camera’s angle of view, as shown in Figure 7. 

The video is recorded with a 16M pixel camera using 25 frames per second (FPS), to be post-processed and analyzed by the use of two open source software package: (a) PIVLAB; a free time-resolved particle image velocimetry (PIV) tool that runs in MATLAB [26] and (b) Tracker; a free video analysis and modeling tool built on the Open Source Physics (OSP) Java framework [27].

### 4.2. Methodology

To perform the experimental validation of the mathematical model, the first step consisted in the measurement of the current circulating in the fluid. The current parameter is an input data required to feed the mathematical model, in order to calculate the flow velocity to be compared with the flow velocity measured with the vision system [28].

**Figure 8:**
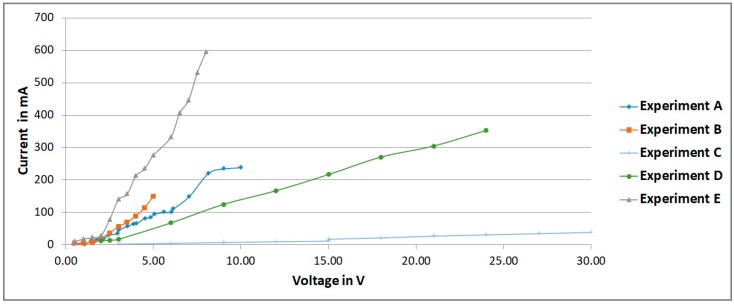
Electric parameters linear relationship for experiments of Table 1.

Table 1 summarizes the different configurations experimented (MHD stirrer geometrical characteristics and solution flow parameters). The measurement of voltage and current are shown in Figure 8 with the purpose to evaluate the conductivity of the fluid by the evaluation of the linear relationship between the applied voltage to the MHD stirrer and the electric current circulating in the fluid.

*Experiment A* was conducted without tracer particles, no fluid movement was detected.

*Experiment B* was conducted without tracer particles, the fluid seemed to be in motion, but it was not verified.

*Experiment C* was conducted using only distilled water and tracer particles, in order to verify if it was possible to have a conductive flow without the secondary effects on the electrodes due to the NaHCO_3_. An electric conductive flow less vulnerable to bubble generation and electrode oxidation was obtained, with particles providing the conditions for analysis with PTV and PIV. The flow presented a regular behavior [2].

*Experiment D* was conducted with tracer particles forming speckles, fluid presented a chaotic movement that produced bubbles [2].

*Experiment E* was conducted with tracer particles forming speckles, fluid presented a chaotic movement that dissolved the speckles [2].

### 4.3. Set up for Mathematical Model Validation

After performing the experiments described in Table 1 to obtain the electric parameters linear relationship, the parameter configuration used in Experiment C was selected. It was selected due to its electrical linearity and due to its regular flow behavior to validate the mathematical model velocity profile, using Particle Image Velocimetry (PIV) and Particle Tracking Velocimetry (PTV). Software’s settings are shown in Section 4.4 and Section 4.5, while results are summarized in Section 4.6.

### 4.4. PIVLAB Toolbox for PIV

**Figure 9:**
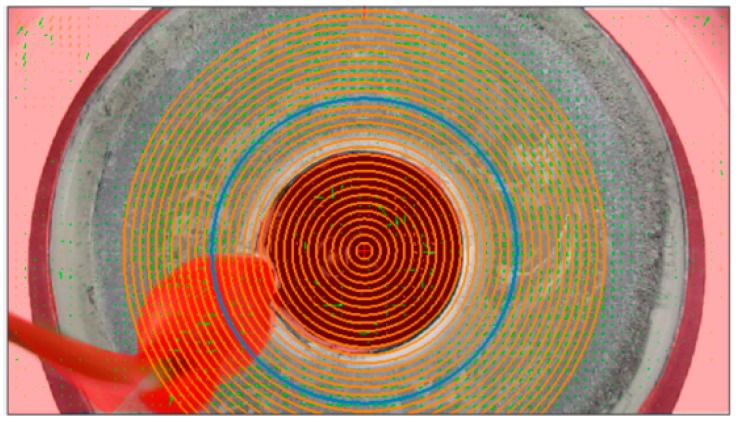
Circle series submitted to velocity measurement.

During experiment C, while the stirrer’s electrical field was induced with 5.0 V, a 41 s video recording was obtained using a 25 FPS camera, resulting in 1025 frames. Due to the current was stabilized at 15 s, the velocity measurement was performed from image frame 901 to image frame 1000. A quantity of 100 images frames with an interval of time *dt* = 0.040 s, were loaded in PIVLAB toolbox, with frames sequence style 901–902, 902–903, 903–904, …, 999–1000. Analysis settings were selected as following: (a) the annular channel region of interest (ROI) was defined masking all not desired areas, (b) contrast-limited adaptive histogram equalization (CLAHE) image pre-processing was selected, with windows size of 20 pixels, (c) PIV settings were selected for Fast Fourier Transformation (FFT) window deformation. Four interrogation cycles with an interrogation area [px] of 128, 64, 32 and 16 for each correspondingly interrogation cycle. Linear interpolation was used for windows deformation, and Gauss 2X3-points were selected as a sub-pixel estimator. The internal diameter size (37.52 mm) and the *dt* interval (40 ms) were used to calibrate the images. Post-processing vector validation was applied to refine velocity limits and eliminate outlier data. The measurements were made in several sections of the channel (through the 360° of the annular channel), in order to confirm stationary flow, which is represented by a zero velocity at a particular fixed point of the fluid. The maximum velocity was identified through the sensing of velocity on circles series, as shown in Figure 9, where the blue circle represents the flow path with the maximum velocity.

### 4.5. Tracker for PTV

A selected particle at r = 26.76 mm from the center (8 mm from the internal cylinder) was tracked in the path of the annular channel, where the maximum flow velocity is presented in the MHD stirrer, according to the result provided by the Galërkin model and proved by the PIVLAB toolbox analysis results. While through the PIVLAB toolbox the velocity was calculated by dividing the distance moved by the time elapsed between two positions; that is, a time between two shots of the camera (frames). Using the Tracker, the position of the particle was registered versus the time along the circle flow path with the maximum flow velocity. The velocity obtained using the PIVLAB toolbox analysis was confirmed at a distance of a ¼ turn and the time elapsed to complete this turn, this measurement is available in the tracker software. The analysis performed with the tracker software in auto-tracking mode gave some level of certainty when the results were compared with Galërkin results but velocities measurement resulted extremely inaccurate due to the software is not the most efficient tool for this application. The particles are so small and the quantity of particle seed in the flow makes impossible for the software to auto-track them, there were selected particles which get lost. As a consequence, the tracking was made manually.

### 4.6. Results

**Figure 10:**
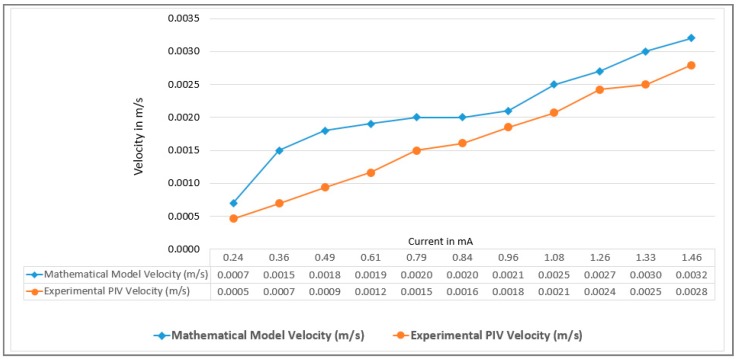
Experimental validation of the mathematical model through the experiment C’s results.

Once the flow velocity measurement method was defined, the experimental validation of the mathematical model has been accomplished. The experiment C has been characterized as shown in Figure 10. It can be seen clearly, that the mathematical model velocity presents a linear behavior, while the experimental velocity has a similar behavior.

**Figure 11:**
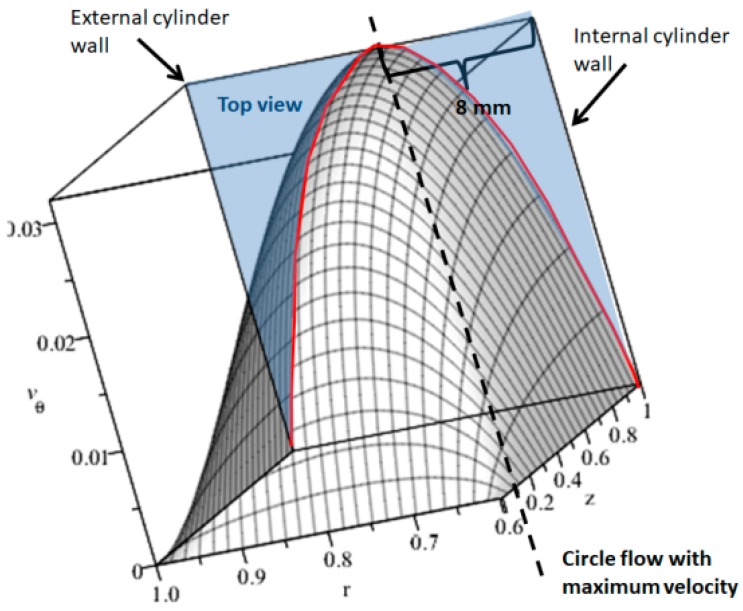
3D Mathematical model velocity profile for experiment C.

**Figure 12:**
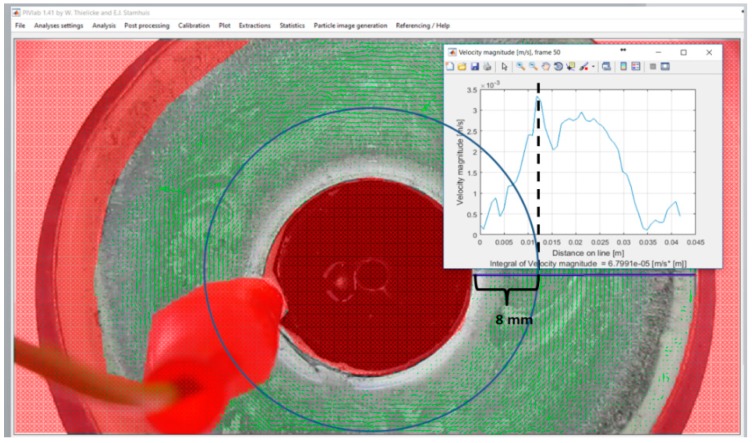
2D PIVLAB toolbox analysis results for experiment C.

**Figure 13:**
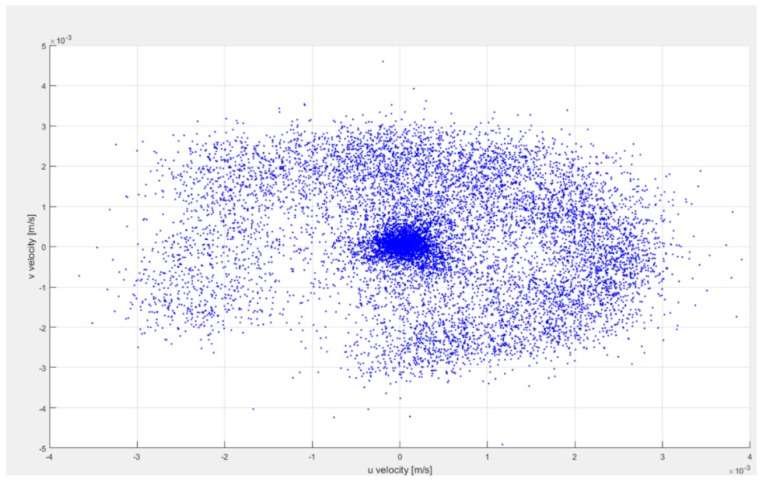
Scatter plot of velocity components u & v, from 2D PIVLAB toolbox analysis results for experiment C.

**Figure 14:**
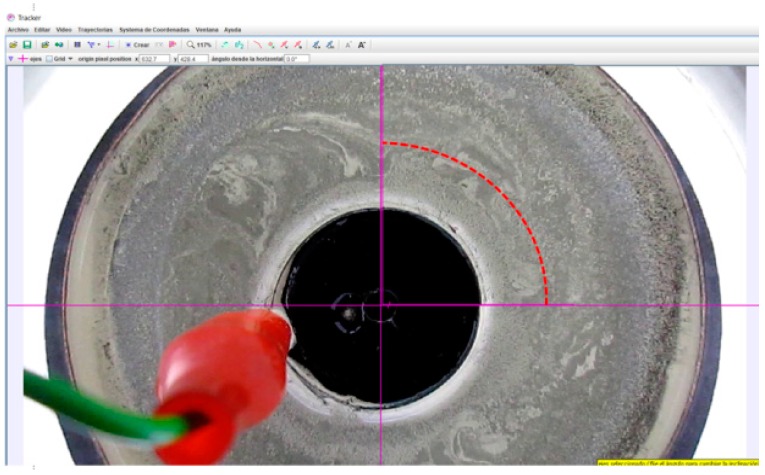
¼ turn tracker analysis of selected particle at r = 26.76 mm from the center (8 mm from the internal cylinder) in the path of the annular duct where the maximum flow velocity is present for experiment C.

As can be observed in Figure 11, a 3D profile has been simulated. In the top view of the MHD stirrer the 2D velocity profile with maximum velocity of 0.0035 m/s can be observed, near to the internal cylinder wall (at 8 mm from the internal cylinder wall). In Figure 12 the 2D velocity profile between cylinders walls, obtained with PIVLAB toolbox is observed; it can be seen that the maximum velocity and its position correspond to model results. Figure 11 and Figure 12 can be compared to clearly observe the difference in the shape of the flow profile between the theory and the experiment results. Although it is true that in the experimental results the shape of the profile is not as perfect as is at of the simulation. It is due to the assumptions that were made in the mathematical model (uniform electric conductivity, uniform magnetic field, low magnetic Reynolds number, low Hartmann number, friction at the walls) and in the sampling resolution of the vision system. Both of them must be taken up again in posterior work. The mathematical model can augment its resolution if more physical variables acting in the mixer are included, but it is not an easy task, in further work more experiments will be realized to identify those parameters affecting the velocity profile shape. Figure 13 represents a scatter plot of the velocity components, which not only include the maximum velocity but all the velocities through the 2D velocity profile. Finally, Figure 14 is a confirmation of model results by the tracker software.

It has been experimentally verified that the maximum velocity and flow rate increase as the slip length parameter grows. The gap between the cylinders has a strong influence in the shape of the velocity profile: the larger the gap, the more asymmetric the profile. The presented analysis results can be used as the starting point for microfluidic mixing studies.

## 5. Conclusions

The MHD phenomenon is a functional option for all micro-applications that require performing a large number of tasks, but the small size of the device is not enough to contain mechanical elements. In this sense, its non-intrusive nature provides an alternative solution to friction, mechanical moving elements breakdowns, undesired vibrations, and noise.

The application of vision systems to analyze fluids behavior in MHD systems is a main source of information for analysis, design of shape, structure, and performance of electromagnetic stirrers for MEMS. In this paper, we theoretically and experimentally analyzed the 3D laminar flow of an electrolyte in an annular open channel driven by a Lorentz force, using a vision system.

The annular duct is formed by two concentric electrical conducting cylinders and limited by an insulating bottom wall. The interaction between a uniform magnetic field along the axial direction and a radial electric current produces a Lorentz force that drives the fluid in the azimuthal direction. The steady flow was solved using a Galërkin method with orthogonal Bessel-Fourier series. A quasi-analytic approximation for velocity was found. Velocity profiles were explored while varying the depth of the channel and the gap between the cylinders in order to show the effect of the walls on the flow configuration.

The methodology and experimentation performed in the design of a vision system for experimental validation of the mathematical model have been detailed. The fluid flow generated in an annular channel, the velocity and its direction was studied based on image processing using PIV and PTV. Mathematical model simulation results were compared with experimental data provided by PIV and PTV technique as well as with the data founded in the scientific literature.

Further work will consider a new combination of NaHCO_3_ and S-HGS tracer particles proportions to obtain an electrolyte flow with a similar conductivity that the obtained in experiment C, but searching to avoid the accumulation of particles in the walls. The MHD stirrer will be modified to introduce laser sheets in order to measure the 2D velocity profiles at different planes, and, in order to experimentally recreate the 3D profile.

Besides, in order to eliminate some issues that have been presented during the experimentation, such as: (a) the accumulation of bubbles along the surface of the electrodes due to water electrolysis, which reduces the current transmission through the electrolyte when the MHD microfluidic system is operating at high voltages; (b) the accumulation of reaction products next to the electrodes due to corrosion, which contaminates the electrolyte and reduces the life of the electrodes. In further research different electrolytes will be evaluated in the search for an electrolyte that does not cause electrode corrosion even when using high current. Also the DC power supply and the permanent magnet will be replaced by an AC power supply and an electromagnet in an experiment. The voltage power supply will be replaced by a current power supply in further experiments.

## Figures and Tables

**Table 1:** Experiment description.

Experiment Number	Geometrical Characteristics					Flow Solution Parameters			Induced Fields	
	$R_{1}$	$R_{2}$	h	η	ε	Distilled Water	Sodium Bicarbonate ${\mathrm{NaHCO}}_{3}$	S-HGS Particles	$B_{0}$	ϕ, dc Voltage
A	18.76 mm	31.44 mm	7 mm	0.6	0.22	100 mL	0.84 g	None	0.1624 T	0.5 V≤Δϕ≤10 V
B	18.76 mm	31.44 mm	7 mm	0.6	0.22	100 mL	8.4 g	None	0.1624 T	0.5 V≤Δϕ≤5 V
C	18.76 mm	31.44 mm	7 mm	0.6	0.22	100 mL	None	0.1 g	0.1624 T	0.5 V≤Δϕ≤30 V
D	18.76 mm	31.44 mm	7 mm	0.6	0.22	100 mL	0.84 g	0.1 g	0.1624 T	2 V≤Δϕ≤24 V
E	18.76 mm	31.44 mm	7 mm	0.6	0.22	100 mL	8.4 g	0.1 g	0.1624 T	0.5 V≤Δϕ≤8 V
